# Stocks and Another *v.* Watson

**Published:** 1901-07-06

**Authors:** 


### The Hospital

A Journal of

The Medical Sciences and Hospital Administration.

Vol. XXX.—No. 771.] Edited by Sir Henry Burdett, K.C.B., and Solomon C. Smith, M.D., M.R.C.P. [July 6, 1901.

### STOCKS AND ANOTHER *v.* WATSON.

An action was tried last week in the King's Bench Division of the High Court before Mr. Justice Bruce and a special jury which very well illustrates the occasional difficulties of surgical practice. The plaintiffs—Mr. Frederick Stocks, surgeon, of the Wandsworth Road, and his partner, Mr. John Crossman—sued the defendant, Watson, who is a publican in Clapham Old Town, for charges amounting to £21 18s., alleged to be due for professional attendance. The defendant disputed the bill and counterclaimed for damages for negligent treatment by the plaintiff Stocks. It appeared from the evidence that the defendant was the subject of a bicycle accident on September 28th, 1899, and that Mr. Stocks was called to him. He found the right shoulder bruised and painful, but, after what he regarded as a sufficient examination, could detect no injury to the joint. He sent the patient a lotion and a sedative and ordered complete rest for the arm, which he bandaged. He described the defendant as a bad patient, who suffered from alcoholism and was restless at night, so that "he" (whether plaintiff or defendant is not quite clear) removed the bandages. The joint soon became badly swollen, in which state it was impossible to examine it; but in the early part of November Mr. Stocks discovered that it was dislocated. An attempt at reduction was unsuccessful, and Mr. Stocks came to the conclusion that it was impossible to replace the bone without the use of an anæsthetic, and that this would be dangerous in the defendant's condition. Mr. Clutton, who was consulted in the following January, confirmed this opinion, and Mr. Stocks soon after ceased to attend. The theory of the plaintiff was that the bone became displaced three or four days after the accident by reason of the defendant's own movements; and that, having once satisfied himself that there was no dislocation, there was nothing to induce him to subject the joint to minute examination again. As far as can be judged from the condensed report, there seems to have been a direct conflict of evidence as to whether the patient was stripped for the first examination. Mr. Clutton, who gave evidence for the plaintiff, was asked by defendant's counsel whether it would not be difficult to detect a dislocation of the shoulder with the coat and waistcoat on, and admitted that it would. Mr. Stocks said that the patient was stripped during his first examination on September 28th, but the defendant swore that he had on a covert coat at the time, and that, although Mr. Stocks afterwards saw him stripped, he did not then make any farther examination, but only put his hand on the shoulder. It is probable that this may have been the turning point of the case, and that the jury believed the plaintiff rather than the defendant; for, after a trial which occupied two days, they found that Mr. Stocks had used ordinary and reasonable skill, and so gave a verdict for the plaintiffs on all the issues. From one point of view, nothing could be more satisfactory; but the fact remains that the unfortunate patient, who took the reasonable and proper course of sending for his doctor as soon as he was hurt, was either then or soon afterwards the subject of a dislocation of the shoulder which was not detected at the time of its occurrence, and which therefore, it may be presumed, will be the cause of some amount of disability for the remainder of his life. Things could hardly have gone worse with him if he had not sent for a doctor at all.

Among the reflections which such a case is calculated to suggest two appear to spring unbidden into prominence; namely, first, would it not have been wise on the part of Mr. Stocks to insist upon an early consultation with a hospital surgeon; and, secondly, was it to the interest of the profession that such an action should be brought? With regard to the former point, it may be presumed that a general practitioner in the Wandsworth Road can hardly have opportunities of seeing any large number of injuries to the shoulder, while such injuries are well known to be often obscure and difficult of diagnosis; so that for such a practitioner to ask, or even to press, for a consultation would have been in no way derogatory to his professional position. The evidence of the defendant, for whatever it may be worth, was that he wished from an early date to call in "a specialist," and that the plaintiff replied, "If you called in fifty specialists, they could do no more for you than I am doing. It would be throwing money away." We fear there is a good deal too much of this attitude in the profession, and in this particular case it is at least possible that any hospital surgeon would have discovered the dislocation as soon as he saw the patient, while it is by no means certain that a skilled anæsthetist would have shrunk from the administration of ether or chloroform. With regard to the action, it is impossible to form an opinion without knowing more of the circumstances than has been disclosed. It might be a perfectly legitimate defence against impending attack by the patient, who may have made some claim for damages against his doctor. Failing this, we greatly doubt whether it was judicious, or whether it could be productive of any advantage to anybody concerned. It may be taken as fairly certain that the amount at issue will not cover the difference between the plaintiffs' taxed costs and their costs as between attorney and client, so that, even if they get all that the law would give them, they will still be out of pocket by the transaction. Sides are sure to be taken, and bad blood created in the locality, where the defendant's crippled arm is likely to be more or less permanently in evidence as a leading fact in the case. There are many positions in life in which it is not desirable to be extreme in asserting or in insisting upon the most undoubted rights; and, if the law of the College of Physicians, by which its fellows and members are prohibited from suing for their fees, were commonly observed in the medical profession, we do not believe that practitioners generally would lose, in money, nearly as much as they would gain, in the public estimation, by such distinct severance of themselves from trade and its customs and associations.

